# Cold Water in Dysentery

**Published:** 1853-10

**Authors:** F. Blade


### *Cold Water in Dysentery.*

BY F. BLADE, M. D.

If it be the accumulated experience of individuals which gives us our *rules* in the practice of medicine, every one ought to contribute his mite, if it be of any value. I am, therefore, prompted to send you a slice of my experience.

Last year I had many cases of dysentery to treat. Some of these "wore the livery" of the ordinary non-malignant variety, and were amenable to the usual remedial means; while others—the majority—were of the epidemic or malignant variety and with surpassing stubbornness "went their ways" heedless of cure, *i. e.*, by the mostly practised methods.

Now, we who have not a reputation to live on after a defeat, cannot well afford—if I may use a sinister expression—to lose many patients consecutively, else we fall into disrepute and straitway lose our practice.

This motive, which was secondary to the heart-felt interest I had in the recovery of my patients, as also this latter motive, caused me to depart from the calomel and opium, etc. etc., *land-marks* in treating the more malignant variety of dysintery. I have now in my mind a case, which conjointly with Dr. Fowler, then my partner, I was called upon to treat. The malady "waxed exceeding sore" from its onset. The griping was positively excruciating; the straining extremely ardent and incessant; the stools exceedingly large, grayish and bloody, containing membranous-like shreds; the pulse was quite frequent and not forcible. This is a rudely sketched outline of the condition of the case as was reported to me to have existed prior to my attendance.—The doctor who was first called had treated the case with calomel and opium, q. s., castor oil and laudanum, as a laxative, once in twenty-four hours, with other adjuvantiæ now passed from memory, for three or four days, at which time I was called to see this case with him.—The above mentioned symptoms were said to be unabated. The pulse was now feeble and about 120; the tongue was covered with a thick brown fur, and dry, the edges were fiery and the whole tongue was dotted over with elevated papillæ—here and there protruding through the fur-coat. The stomach was so excessively irritable that it would scarcely retain a tea-spoonful of water. I suggested an enema consisting of a *strong* solution of nitrate of silver, which was twice or thrice repeated during the ensuing twenty-four hours. Also camphor spirits and oil of turpentine, equal parts, to be applied almost hot, to the abdomen. It was of no use. The disease increased in severity. We looked upon the mortal issue as being but a few hours in advance of us. Here was our extremity, and *cold water* was the straw caught at. What miraculous buoyancy there was in that dernier resort! We left off medicine entirely—little use was it when none would be retained by the stomach—and determined to try cold water. We wrapped the patient in a cold wet sheet and thereupon—having previously passed a stool every ten or fifteen minutes—he lay one hour and a half, without having desire to go to stool. At the end of this time, the surface almost glowed with warmth, and there was the moisture of sweat about the face and neck. The patient was then wiped dry with coarse towels and placed in a dry bed. This operation was thenceforward repeated every five or six hours for the next five days, after which time it was only used once or twice in twenty-four hours for two or three days longer. Instead of the warm fomentations, which had been constantly applied, cloths wrung out of cold water were frequently repeated to the abdomen. As enemas we used cold water, simply—eight or ten ounces immediately after every evacuation. In every case in the treatment of which we used cold water injections it was found to be important that it should be administered immediately subsequent to every stool. They were borne without distress, and much longer. I ought, also, to mention that after the first day we used the cold Sitz-bath of the hydropathists. From the commencement of this treatment the irritability of the stomach was entirely appeased; the stools became less and less in frequency and of a more natural appearance and consistence. As a diet, as well as an auxiliary to the treatment, we ordered the animal broths well salted.

Several other cases I have in my mind of a like character with the above. With the exception of one, none of them were so violently attacked. That case being of a more robust habit, the disease did not succumb so rapidly. I commenced treating with calomel, ipecac., and *one grain* of morphine every three hours—at the end of twenty-four hours, giving a castor oil laxative—warm fomentations to the abdomen; enemas of cold water and laudanum. This course was kept up with more or less modification until the expiration of a week. I was not flattered by the progress my patient had made for the better. I then resorted to the water treatment—carrying it out as in the first instance. Upon the first using of the wet sheet the bowels were quieted two hours—having been previously moved as often as from fifteen to thirty minutes. The patient kept right on improving—steadily, yet, I confess, slowly. It was gratifying to see the complete relief from the excrutiating tormina and tenesmus which followed the "wet-sheet packing."

In this case I used, as often as once in four hours, the turpentine emulsion, strongly charged with laudanum. I also occasionally ordered laudanum in the injections.

In many cases, the cold, wet bandage, and cold water injections, were used as auxiliaries to other treatment, and with a highly gratifying effect.

I am so thoroughly convinced of the powerful efficacy of cold water in the treatment of dysentery that I do not hesitate to say I regard it as one of the chief remedies for combating that formidable disease.

Dr. Bennett, of this place, a practitioner of many years standing, and a correct observer, after being repeatedly disappointed by depending upon the ordinary remedies alone, is, upon fair trial in many instances, enthusiastic in his confidence in cold water as a powerful auxiliary in treating dysentery.

It would be absurd to argue a general rule from such limited experience, yet its effects have been so highly gratifying in the hands of many practitioners, that it is hard to resist the conviction that cold water deserves a more honorable place among the therapia of dysentery than it has hitherto obtained.—*North-western Med. and Surg. Journal.*

